# Damage control surgery – experiences from a level I trauma center

**DOI:** 10.1186/s12891-017-1751-6

**Published:** 2017-09-11

**Authors:** Bernhard Gasser, Thomas M. Tiefenboeck, Sandra Boesmueller, Danijel Kivaranovic, Adam Bukaty, Patrick Platzer

**Affiliations:** 1 Department of Trauma Surgery, Medical University of Vienna, Waehringerguertel 18-20, A-1090 Vienna, Austria; 2 Division of General Anaesthesia and Intensive Care Medicine, Medical University of Vienna, Vienna, Austria; 3 Section for Medical Statistics, Center for Medical Statistics, Informatics, and Intelligent Systems, Medical University of Vienna, Vienna, Austria

**Keywords:** Damage control surgery, Intramedullary nailing, External fixation, Outcome, Retrospective study

## Abstract

**Background:**

There is still no evidence in literature for damage control orthopaedics (DCO), early total care (ETC) or using external fixation solely in fractures of the long bones in multi-system-trauma. The aim of this study was to determine parameters influencing the choice of treatment in clinical routine (DCO, ETC, or EF) in femoral or tibial shaft fractures in combination with multi-system-trauma, severe soft tissue damage or both.

**Methods:**

Data of 236 patients with 280 fractures of long bones of the lower extremities treated at a level I trauma center were analysed. Clinical parameters on arrival (age, sex [m/f], ISS, fracture site [femur/tibia], soft tissue damage [closed or open fractures according to the Gustilo-Anderson classification], pulmonary injury [yes/no]) were collected and analysed whether they influence the choice of upcoming treatment (DCO/ETC/EF).

**Results:**

Our findings showed that high ISS and severe soft tissue damage (grade III) significantly correlated with DCO. High ISS, old age, female sex and fracture site (tibia) correlated with EF. This group of sole use of external fixation had highest rate of complications, 69% were associated with at least one complication.

**Conclusion:**

Severely injured patients are treated significantly more often with DCO or EF. The presence of higher ISS (≥16) and of type III open fractures increased the use of DCO. However, ISS, fracture-site, patient’s age, type III open fractures or sex (female) increased the use of EF compared to ETC.

## Background

Since 1970, intramedullary nailing (IMN) has yielded improved clinical and functional results in trauma surgery [1–4], and has become a commonly-accepted treatment option for long-bone fractures. However, problems associated with IMN have been mentioned in literature [5–7], and might influence the decision to perform this procedure [8, 9]. Either way, a surgeon tasked with treating severe open fractures of the femur or tibia, or with treating multisystem trauma involving fractures of the femur or tibia, is confronted with the same question: Would it be better to perform IMN, or should (temporary) external fixation be implemented?

Early stabilisation of femoral shaft fractures provides benefits for patients with multi-system-trauma [10]. However, there is evidence in the literature suggesting that subsequent surgery after severe trauma acts as a “second hit” to the patient’s system, effectively serving to worsen his or her situation [10, 11]. This has to be taken in account, especially, when intramedullary osteosynthesis within hours of trauma (approach of ETC) is considered. Therefore, the timing of IMN in patients with multisystem trauma must be discussed – and carefully considered. The Injury Severity Score (ISS) was used to measure the overall trauma burden. ISS is 0 in uninjured persons and increases to a maximum, per definition, of 75. Probability of death increases with higher ISS [12].

Based in part upon the concept of avoiding this “second hit”, “Damage Control Orthopaedics” (DCO) has become a popular procedure in severely injured patients – as it serves to provide early stabilisation, while also taking advantage of the benefits of IMN [13–15]. Within this concept, the initial surgical trauma can be reduced as intramedullary manipulation is avoided by using external fixation. After recovery of the patient, most likely on an intensive care unit, external fixation is removed and a nail is implemented to be in favour of stable osteosynthesis.

Despite its popularity, though, the literature provides little information as to exactly which patients would benefit from DCO or not [16].

The aim of this retrospective study was to identify parameters that influence the clinical choice for either Damage Control Orthopaedics (DCO), Early Total Care (ETC) or External Fixation (EF) and further to investigate whether these parameters are predicting factors concerning patient’s prognosis.

## Methods

Between 1992 and 2010, the database of the Department of Trauma Surgery contained more than 5500 patients who had sustained femoral or tibial fractures. Included for study were all patients (1) with fractures of the tibial or femoral shaft, associated with Injury Severity Score [12] (ISS) ≥16, or (2) with open fractures (grade II or higher according to the Gustilo/Anderson classification [17]) of the tibial or femoral shaft and, (3) whose initial surgical treatment was either IMN or external fixation and (4) whose age was ≥18 years. Exclusion criteria were as follows: (1) Primary admission and treatment beyond a primary survey in another hospital, (2) death before operative treatment, (3) index fractures in bones with pre-existing osteosynthesis or prosthesis, (4) ISS <16 if index-fracture was closed or open grade I, (5) age < 18 years and (6) incomplete data set (e.g., transfer to another hospital, loss to follow-up or death after release from hospital). Isolated fractures without significant soft tissue damage (i.e. closed fractures or grade I open fractures) were not addressed in this analysis because these fractures are usually treated by ETC at our institution. However, fractures with minor soft tissue damage (closed or open grade I) associated with ISS ≥16 were considered as being a part of multi-system-trauma and were therefore included.

We identified 236 patients with 280 fractures meeting our inclusion criteria. All database files and medical records were retrospectively reviewed for clinical, functional and demographic data. Clinical parameters were collected, including ISS [12], fracture site (e.g., femoral or tibial shaft), fracture classification (e.g., closed, or type I, II or III according to the Gustilo-Anderson classification), radiological evidence of lung contusion, pneumothorax, haematothorax or pneumo-haematothorax (yes/no), sex and age. Furthermore, presence of traumatic brain injury (TBI) (yes/no) was recorded for descriptive statistics.

In patients with suspected additional lung injury CT-scans were obtained to identify or exclude thoracic injury. These scans were evaluated by trauma surgeons together with radiologists experienced in polytrauma diagnostics.

To evaluate outcome, we assessed the presence of severe complications. The development of ARDS was graded as a severe complication, as was the presence of any local incident requiring surgical treatment. Such incidents were as follows: (1) infection requiring surgery, (2) delayed union (the absence of bony healing 4 months after injury) or non-union (the absence of bony healing 6 months after injury), (3) hardware malfunction including pin loosening, malposition and breakage of osteosynthetic material, (4) re-fracture and (5) amputation. If a situation would have necessitated surgery, and this surgery was not performed – be it due to a patient withholding consent, or due to his or her generally poor condition – the incident was still graded as a severe complication.

For final analysis, patients were divided into 3 groups according to their treatment modality: (1) IMN as the first procedure of stabilization (ETC), (2) external fixation as definitive fracture stabilisation (EF), and (3) the combination of first installing an external fixator, with the later removal of the fixator and implantation of an intramedullary nail (DCO). Although external fixation was also used within the DCO-group, the shortcut “EF” denotes when external fixation was solely used.

Patients matching the inclusion criteria but not surviving hospital stay were incorporated in the demographic description – but they were not included for statistical analysis.

Prior to investigation, this study was approved by the corresponding Institutional Review Board.

### Statistical analysis

The median with interquartile range (IQR) was calculated for quantitative variables, whereas qualitative variables were described using absolute and relative frequencies. The allocation of patients to their respective treatments was not randomised; therefore, a generalized propensity score adjustment for pre-treatment differences was applied to assess the complication rates of ETC, EF and DCO [18].

The propensity score is defined as the probability to receive a treatment given the observed pre-treatment variables. At first, the propensity score was estimated by a multinomial logistic regression model of the form:

Treatment ~ ISS + classification of open fractures + thoracic injury + fracture site + age + sex.

The value of the propensity score potentially ranges from 0 (indicating a probability of using the treatment given being 0% in a situation defined by the pre-treatment variables) to 1 (indicating a probability of using the treatment given being 100% in a situation defined by the pre-treatment variables).

We then used logistic regressions to model the relationship between complications and log odds of the propensity score for each of the three observed treatment groups separately. Finally, the estimated model parameters were used to predict the risk of complications in all patients, observed and unobserved, for each treatment group. The adjusted complication rates are the average overall predicted values of each treatment group. Parameters of the multinomial regression were tested by z-tests. 95% confidence intervals for the adjusted complication rates and odds ratios were calculated by bootstrap with 10,000 replications. *P*-values <0.05 were considered to be statistically significant. All calculations were performed in the R 3.0.2 statistical computation environment.

## Results

Two hundred thirty-six patients with 280 fractures matched the inclusion criteria, of which 210 patients with 244 fractures survived hospital stay, and therefore were used to analyse complication rates and propensity score. Overall, 201 fractures were seen in males (72%) and 79 in females (28%) with a median age of 34 years (IQR: 25–48.5; range: 18–96). 170 tibial fractures (61%) and 110 femoral fractures (39%) were observed. One hundred twelve fractures (40%) were closed or open type I, 80 (29%) were open type II and 88 (31%) were open type III. Ninety-four fractures (34%) were accompanied by lung injury. The mean length of intensive care unit stay was 12 days (SD: 14; range: 0–100; median: 8). The median ISS at time of admission was 18 (IQR: 9–22; range: 9–66). The following mechanisms of trauma were observed: 206 traffic accidents (74%), 39 falls from a height more than 3 m (14%), 17 simple falls from a height less than 3 m (6%), and in 18 cases (6%) other causes were identified. One hundred twenty-nine fractures (46%) were treated with ETC, 107 fractures (38%) with EF and 44 fractures (16%) with DCO. Detailed overview of patient’s demographics is shown in Table 1.

**Table 1:** Demographics by treatment groups including patients who died

	ETC	DCO	EF
ISS (median)	17	22	19
Age (mean)	35,7	31,8	43,4
Fracture type			
Closed/Open I°	37,2% (*n* = 48)	54,5% (*n* = 24)	37,4% (*n* = 40)
Open II°	44,2% (*n* = 57)	11,4% (*n* = 5)	16,8% (*n* = 18)
Open III°	18,6% (*n* = 24)	34,1% (*n* = 15)	45,8% (*n* = 49)
Pulmonary injury			
No	72,9% (*n* = 94)	45,5% (*n* = 20)	67,3% (*n* = 72)
Yes	27,1% (*n* = 35)	54,5% (*n* = 24)	32,7% (*n* = 35)
Intracranial injury			
No	72,9% (*n* = 94)	59,1% (*n* = 26)	71,0% (*n* = 76)
Yes	27,1% (*n* = 35)	40,9% (*n* = 18)	29,0% (*n* = 31)
Fracture site			
Femur	40,3% (*n* = 52)	68,2% (*n* = 30)	25,2% (*n* = 27)
Tibia	59,7% (*n* = 77)	31,8% (*n* = 14)	74,8% (*n* = 80)
Side			
Right	53,5% (*n* = 69)	47,7% (*n* = 21)	44,9% (*n* = 48)
Left	46,5% (*n* = 60)	52,3% (*n* = 23)	55,1% (*n* = 59)
Sex			
Male	78,3% (*n* = 101)	75,0% (*n* = 33)	62,6% (*n* = 67)
Female	21,7% (*n* = 28)	25,9% (*n* = 11)	37,4% (*n* = 40)
Total	100% (*n* = 129)	100% (*n* = 44)	100% (*n* = 107)

° = grade

### Treatment strategy in relation to clinical parameters

Using the multinomial logistic regression, we analysed the influence of pre-treatment parameters on treatment decision. ETC was the reference group in the analysis. Detailed results are presented in Table 2. Severely injured patients were significantly more often treated with DCO or EF. The odds ratios for ISS and type III open fractures were highly significant in both treatment groups; however, type II open fractures did not influence the decision-making. Only in the EF group were the odds ratios for age, fracture site and sex significantly greater than 1. This means that the tendency towards EF increases with age, a fracture of the tibia is more often treated with EF than a fracture of the femur, and that women were more frequently treated with EF than their male counterparts. Decision-making was not dependent upon pulmonary injuries.

**Table 2:** Odds ratios and p-values of the multinomial logistic regression. ETC is the reference group. OR denotes the odds ratio, and CI the 95% confidence interval

Predictor variable	DCO		EF	
	OR (CI)	*p*-value	OR (CI)	*p*-value
ISS	1.13 (1.06–1.20)	0.0002*	1.12 (1.06–1.19)	<0.0001*
Age	0.99 (0.96–1.02)	0.3252	1.03 (1.01–1.05)	0.0017*
Fracture type				
Closed/Open I°	1 (ref.)		1 (ref.)	
Open II°	0.65 (0.20–2.12)	0.4696	0.65 (0.27–1.55)	0.3329
Open III°	5.32 (1.85–15.26)	0.0019*	6.28 (2.55–15.42)	<0.0001*
Pulmonary injury				
No	1 (ref.)		1 (ref.)	
Yes	1.49 (0.63–3.54)	0.3623	0.88 (0.42–1.86)	0.7402
Fracture site				
Femur	1 (ref.)		1 (ref.)	
Tibia	0.60 (0.26–1.41)	0.2418	2.45 (1.23–4.85)	0.0103*
Sex				
Male	1 (ref.)		1 (ref.)	
Female	1.30 (0.53–3.17)	0.2697	2.10 (1.07–4.10)	0.0304*

* indicates the *p*-value to be significant

° = grade

In our patients, an increasing propensity score led to a decreased probability of complication, which also applied to all treatment groups. However, all of these trends failed to reach significance (*p* = 0.2650 in ETC group; *p* = 0.1463 in DCO group; *p* = 0.5737 in EF group).

### Complication rates

At least one complication was present in 33% of the fractures during the follow-up period. The highest complication rate was seen in the EF (external fixation only) group (69% fractures were associated with at least one complication; confidence interval: 58%–78%). Significantly lower complication rates were seen in DCO group (23% with at least one complication; confidence interval: 12%–36%) and ETC group (20% with at least one complication; confidence interval: 14%–28%). Detailed overview of Percentage and numbers of complications within treatment groups is presented in Table 3.

**Table 3:** Percentage and numbers of all complications within treatment groups

	ETC (*n* = 129)	DCO (*n* = 44)	EF (*n* = 107)
Infection	3,9% (*n* = 5)	9,1% (*n* = 4)	12,1% (*n* = 13)
Delayed Union/Non-union	9,3% (*n* = 12)	11,4% (*n* = 5)	22,4% (*n* = 24)
Malfunction (Loosening, Malposition, Hardware Failure)	3,1% (*n* = 4)	6,8% (*n* = 3)	27,1% (*n* = 29)
Re-Fracture	0	0	1,9% (*n* = 2)
Amputation	0	0	6,5% (*n* = 7)
ARDS	3,9% (*n* = 5)	4,5% (*n* = 2)	1,9% (*n* = 2)
Died	3,9% (*n* = 5)	0	28,0% (*n* = 30)

**Fig. 1:**
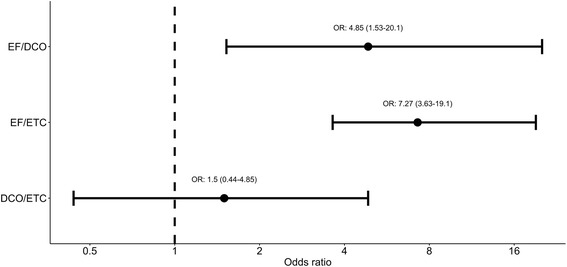
Odds ratios for adjusted complication rates and 95% confidence intervals. Estimates with confidence intervals greater than 1 are statistically significant

The observed complication rates were 20%, 23% and 69% for ETC, DCO and EF, respectively. Adjusting pre-treatment differences via the propensity score regression adjustment method did not change the complication rates considerably; the adjusted complication rates with 95% confidence interval were 22% (19%–25%), 30% (23%–36%) and 68% (63%–72%) for ETC, DCO and EF, respectively. Patients treated with EF had a significantly higher risk of complications, but there was no significant difference between the DCO and ETC groups (Fig. 1).

## Discussion

Summarizing our results, we could show that more severely injured patients (high ISS) were significantly more often treated with DCO or EF. This corresponds with the international trend of not performing IMN in the context of multi-system-trauma. The presence of pulmonary injury did not change the choice of treatment in our study cohort.

The ISS parameter (≥16) and the presence of type III open fractures increase the use of DCO when compared to ETC. However, ISS, type III open fractures, tibial fracture, increasing patient age, or sex (female) increased the use of EF compared to ETC. This might be caused by a higher rate of comorbidities in elderly patients, the poor soft tissue mantle along the tibia and the fact that women presented as being older than their male counterparts in our study cohort.

This result might also be biased by the higher occurrence of femoral shaft fractures in young male patients after high-energy trauma, a patient population which might possibly be more physically capable of tolerating ETC.

We observed a high propensity score predicting lower complication rates. A high propensity score indicates multiple parameters favouring the given treatment. As in these cases the complication rates decrease, the overall decision-making seems to follow correct considerations, however, this trend fails to reach significance in the present study. This trend suggests that the choice of treatment was correct, and our data favour the idea that damage control surgery needs to be performed in selected patients in order to reduce the risk of severe complications.

The high complication rate of 69% in the EF group indicates that a conversion to intramedullary nail should be performed. However, the reason not to do so in our collective, was poor general condition and/or severe soft tissue damage, which would have not allowed further surgery.

The presence of type II open fractures did not change the choice of treatment significantly, although there was a trend towards initial stabilization via IMN. Open fractures grade III significantly lead more often to EF, compared to ETC. In these cases of severe soft-tissue damage no significance was found between the usage of DCO and ETC. In the literature, the majority of articles present type III open fractures as being treated exclusively by EF and initial nailing [6, 8, 19–21]. The use of staged treatment for these fractures was rarely promoted [22, 23].

In 2000, Scalea et al. [24] presented a retrospective study in which they compared the outcome of patients with femur fractures initially treated with IMN to those initially treated with EF. All patients with EF received secondary IMN, except those patients who either did not survive, required amputation or exhibited skeletal immaturity. Patients treated with EF were injured more severely than those who were treated with IMN. However, the two groups did not differ significantly in terms of demographics or outcomes. The use of EF as a bridging procedure is presented as being safe in patients with multi-system-trauma [24]. Similar results have been published confirming this [15, 16, 25]. Further articles have observed a decrease in inflammatory response in patients who received DCO, as compared to patients who received ETC.

Harwood et al. compared DCO and ETC – specifically in regards to systemic inflammatory response – in severely injured patients (New Injury Severity Score (NISS) ≥20) with femoral shaft fractures. In this study the DCO group had suffered more severe injuries. Nevertheless, the systemic inflammatory response syndrome (SIRS) score was significantly lower in this group after initial stabilization. The same proportions were seen when comparing DCO patients undergoing conversion to IMN and ETC patients after initial surgery [26]. In a prospective trial performed by Pape et al., patients with femoral fractures and an ISS >16 were assigned randomly to either a DCO- or ETC-protocol. In pre-, peri- and postoperative blood samples, the proinflammatory cytokines IL-6 and IL-8 were measured. Mirroring the aforementioned study, these interleukins rose significantly after IMN, but did not rise during stabilization via EF or after conversion [27]. This means that it is possible to reduce the systemic inflammatory response at any time by performing a staged protocol.

Although literature provides evidence that DCO is an effective way to treat severely injured patients with fractures of the long bones, even review articles fail to clearly define the conditions in which DCO should be performed [28–30]. Surprisingly, none of the previously mentioned articles on DCO takes into account any kind of soft tissue damage.

Type II and III open fractures are challenging injuries which are normally treated with ETC or EF [6, 8, 19–21]. For this pattern of injuries, the efficacy of a staged protocol combining EF and IMN has been reported [22, 23].

This study has several limitations. Most of them are the result of its retrospective design: There was no strict protocol mandating a particular kind of primary stabilisation for the fractures presented. Hence, it was a case-by-case decision whether to do ETC via IMN or to apply (temporary) external fixation. There was also no strict protocol dictating whether to perform secondary intramedullary nailing (DCO) and if this was indeed performed, this was an individual decision for each patient. However, our study presents the experience of a large level I trauma center, reporting for the first time parameters influencing the choice of treatment in severely injured patients.

## Conclusion

We found that severely injured patients are treated significantly more often with DCO or EF. The presence of a high ISS and the presence of type III open fractures increase the use of DCO. However, ISS, tibial fracture, older patient age, type III open fractures or sex (female) increased the use of EF compared to ETC. However, using EF as sole treatment was associated with a higher rate of complications.

## Abbreviations

DCO: Damage control orthopaedics
EF: External fixator
ETC: Early total care
IL: Interleukin
IMN: Intramedullary nailing
ISS: Injury severity Score
NISS: New Injury Severity Score
SIRS: Systemic inflammatory response syndrome
TBI: Traumatic brain injury

## References

[1] Kamdar BA, Arden GP (1974). Intramedullary nailing for fractures of the femoral shaft. Injury.

[2] Christie J, Court-Brown C, Kinninmonth AW, Howie CR (1988). Intramedullary locking nails in the management of femoral shaft fractures. J Bone Joint Surg Br.

[3] Johnson K, Tencer AF, Sherman MC (1987). Biomechanical factors affecting fracture stability and femoral bursting in closed Intramedullary nailing of femoral shaft fractures, with illustrative case presentations. J Orthop Trauma.

[4] Sojbjerg JO, Eiskjaer S, Moller-Larsen F (1990). Locked nailing of comminuted and unstable fractures of the femur. J Bone Joint Surg Br.

[5] Bråten M, Terjesen T, Rossvoll I (1995). Femoral shaft fractures treated by intramedullary nailing. A follow-up study focusing on problems related to the method. Injury.

[6] Noumi T, Yokoyama K, Ohtsuka H, Nakamura K, Itoman M (2005). Intramedullary nailing for open fractures of the femoral shaft: evaluation of contributing factors on deep infection and nonunion using multivariate analysis. Injury.

[7] Schemitsch EH, Kowalski MJ, Swiontkowski MF, Senft D (1994). Cortical bone blood flow in reamed and unreamed locked intramedullary nailing: a fractured tibia model in sheep. J Orthop Trauma.

[8] Agrawal A, Chauhan VD, Maheshwari RK, Juyal AK (2013). Primary nailing in the open fractures of the tibia-Is it worth?. J Clin Diagn Res JCDR.

[9] Pape H-C (2007). Impact of the method of initial stabilization for femoral shaft fractures in patients with multiple injuries at risk for complications (borderline patients). Ann Surg.

[10] Brundage SI, McGhan R, Jurkovich GJ, Mack CD, Maier RV (2002). Timing of femur fracture fixation: effect on outcome in patients with thoracic and head injuries. J Trauma.

[11] Recknagel S (2013). Conversion from external fixator to intramedullary nail causes a second hit and impairs fracture healing in a severe trauma model. J Orthop Res.

[12] Baker SP, O’Neill B, Haddon W, Long WB (1974). The injury severity score: a method for describing patients with multiple injuries and evaluating emergency care. J Trauma.

[13] Steinhausen E (2014). A risk-adapted approach is beneficial in the management of bilateral femoral shaft fractures in multiple trauma patients: an analysis based on the trauma registry of the German Trauma Society. J Trauma Acute Care Surg.

[14] Harvin JA (2012). Early femur fracture fixation is associated with a reduction in pulmonary complications and hospital charges: a decade of experience with 1,376 diaphyseal femur fractures. J Trauma Acute Care Surg.

[15] Caba-Doussoux P, Leon-Baltasar JL, Garcia-Fuentes C, Resines-Erasun C (2012). Damage control orthopaedics in severe polytrauma with femur fracture. Injury.

[16] Andruszkow H (2013). Surgical strategies in polytraumatized patients with femoral shaft fractures - comparing a German and an Australian level I trauma centre. Injury.

[17] Gustilo RB, Anderson JT (1976). Prevention of infection in the treatment of one thousand and twenty-five open fractures of long bones: retrospective and prospective analyses. J Bone Joint Surg Am.

[18] Feng P, Zhou X-H, Zou Q-M, Fan M-Y, Li X-S (2012). Generalized propensity score for estimating the average treatment effect of multiple treatments. Stat Med.

[19] Henley MB (1998). Treatment of type II, IIIA, and IIIB open fractures of the tibial shaft: a prospective comparison of unreamed interlocking intramedullary nails and half-pin external fixators. J Orthop Trauma.

[20] Webb LX, Bosse MJ, Castillo RC, MacKenzie EJ (2007). Analysis of surgeon-controlled variables in the treatment of limb-threatening type-III open tibial diaphyseal fractures. J Bone Joint Surg Am.

[21] Inan M, Halici M, Ayan I, Tuncel M, Karaoglu S (2007). Treatment of type IIIA open fractures of tibial shaft with Ilizarov external fixator versus unreamed tibial nailing. Arch Orthop Trauma Surg.

[22] Antich-Adrover P, Martí-Garin D, Murias-Alvarez J, Puente-Alonso C (1997). External fixation and secondary Intramedullary nailing of open tibial fractures a randomised, Prospective Trial. J Bone Joint Surg Br.

[23] Dar GN, Tak SR, Kangoo KA, Dar FA, Ahmed ST (2009). External fixation followed by delayed interlocking intramedullary nailing in high velocity gunshot wounds of the femur. Ulus Travma Ve Acil Cerrahi Derg Turk J Trauma Emerg Surg.

[24] Scalea TM (2000). External fixation as a bridge to Intramedullary nailing for patients with multiple injuries and with femur fractures: damage control orthopedics. J Trauma-Inj Infect.

[25] Morshed S (2009). Delayed internal fixation of femoral shaft fracture reduces mortality among patients with multisystem trauma. J Bone Joint Surg Am.

[26] Harwood PJ, Giannoudis PV, van Griensven M, Krettek C, Pape H-C (2005). Alterations in the systemic inflammatory response after early total care and damage control procedures for femoral shaft fracture in severely injured patients. J Trauma.

[27] Pape H-C (2003). Impact of intramedullary instrumentation versus damage control for femoral fractures on immunoinflammatory parameters: prospective randomized analysis by the EPOFF Study Group. J Trauma.

[28] Hildebrand F, Giannoudis P, Kretteck C, Pape H-C (2004). Damage control: extremities. Injury.

[29] Flierl MA (2010). Femur shaft fracture fixation in head-injured patients: when is the right time?. J Orthop Trauma.

[30] Rixen D (2005). Evaluation of criteria for temporary external fixation in risk-adapted damage control orthopedic surgery of femur shaft fractures in multiple trauma patients: ‘evidence-based medicine’ versus ‘reality’ in the trauma registry of the German Trauma Society. J Trauma.

